# A Global Health Alert on the Re‐Emergence of Mpox a Viral Zoonotic Illness Its Prevention and Management: A Narrative Review

**DOI:** 10.1002/hsr2.72560

**Published:** 2026-05-27

**Authors:** Muslim Bin Aqeel, Asif Hanif, Shahzad Ahmad, Fatima Iftikhar Shah, Benish Javed, Shazia Iqbal

**Affiliations:** ^1^ Biomedicine Program, School of Health Sciences, Health Campus Universiti Sains Malaysia Kubang Kerian Kelantan Malaysia; ^2^ Department of Biostatistics, Faculty of Medicine Sakarya University Sakarya Turkey; ^3^ Faculty of Medicine and Health Sciences The University of Buckingham Buckingham UK; ^4^ University Institute of Medical Lab Technology, Faculty of Allied Health Sciences The University of Lahore Lahore Pakistan

**Keywords:** antiviral treatments, clinical features, epidemiology, infection control, monkeypox virus (MPXV)

## Abstract

**Background and Aims:**

Monkeypox virus (MPXV) is gaining attention in the global health community due to its potential for human transmission and similarities to the well‐known smallpox virus. Mpox was first detected in the 1970s and mostly affects Central and West Africa. However, it has caused rare outbreaks in isolated regions worldwide, indicating a possible global health impact.

**Methodology:**

In this review, we will look at several elements of mpox, such as its epidemiological patterns, clinical symptoms in infected individuals, and contemporary diagnostic and preventative techniques. A large portion of this review is also devoted to assessing the efficacy and safety of investigational antiviral medicines like cidofovir and brincidofovir. These assessments are based on controlled experiments conducted in laboratory settings and investigations on animal models.

**Results:**

So far, the medical profession lacks antiviral drugs, particularly intended to combat mpox. However, research has yielded promising outcomes in the usage of investigational medications such as cidofovir and brincidofovir. In the absence of targeted antiviral therapies, mpox is primarily managed by supportive care. This involves attempts to alleviate symptoms, keep hydration and electrolyte balance, and adopt effective infection control techniques.

**Conclusion:**

This review provides an informative and thorough examination of mpox, emphasizing its importance and possible hazards in both public health and therapeutic settings. The lack of specialized antiviral therapies for mpox is a huge gap in our medical arsenal. However, continuous research into new medications provides a glimpse of optimism.

## 1 Introduction

During the ongoing COVID‐19 pandemic, there has been growing alarm among public health authorities regarding a recent epidemic of monkeypox virus, which is raising concerns about a potential new hazard [1, 2]. The MPVX is a zoonotic pathogen that causes a rash akin to that of smallpox [3]. The virus was given the name monkeypox virus due to its near resemblance to other recognized poxviruses [4]. The initial documented instance of human monkeypox infection may be traced back to the year 1970 a 9‐month‐old newborn from the second largest country in Africa with the fourth largest population was affected. Currently, there is a mounting apprehension regarding the occurrence of mpox epidemics in the Western Hemisphere. Human mpox is an infrequent zoonotic viral disease that predominantly occurs in the central and western regions of Africa. Significantly, this illness garnered considerable attention as a result of the outbreak that occurred within the United States from 2003 to 2004 [5].

Although the historical prevalence of the monkeypox virus in traditionally endemic places for an extended period of time, research pertaining to mpox has suffered from neglect and insufficient funding. As of early May 2022, there have been more than 3000 documented cases of monkeypox virus infections across 50 nations in five different regions. This significant increase in cases has led the World Health Organization to classify mpox as a “developing threat of moderate public health concern” on 23 June, 2022 [6]. The transmission of the monkeypox virus occurs through the exchange of substantial respiratory secretions, near or close interaction with skin lesions, and potentially contaminated inanimate objects. There is a lack of clear evidence about the transmission of sexually transmitted infections through semen or through vaginal secretions [7]. Thus far, the prevailing transmission patterns have exhibited a disproportionate impact on individuals identifying as homosexual or bisexual, as well as those engaging in sexual activities with other men. This observation suggests an escalation in transmission rates within interconnected sexual networks [8].

The monkeypox virus is classified as an Orthopoxvirus, belonging to the Poxviridae family of zoonotic viruses. The viruses belonging to the family Poxviridae are distinguished by their substantial dimensions, contained architecture, and possession of double‐stranded DNA. These viruses are commonly observed in a state of separation from a wide range of animal species. Poxviruses primarily utilize rodents, rabbits, and non‐human primates as their hosts, which might sporadically facilitate the transmission of these viruses to people, thereby fostering human‐to‐human transmission [9]. Both the *Entomopoxvirinae* and the *Chorodopoxvirinae* families are subfamilies of the Poxviridae family in the taxonomic classification system. The classification of viruses into subfamilies is determined by whether or not the virus may infect insects, as in the instance of the *Entemopovirinae*, or vertebrates, as in the case of the *Chorodopoxvirinae* [10, 11].

In order to facilitate the timely identification of mpox patients, the authorities in charge of the general health have implemented stringent protocols and case criteria [12]. Disease transmission can be halted, and people at high risk for consequences can be identified and treated sooner if they are identified early [13]. Although this is important, it has been slowed by the fact that the majority of the information we need to understand human disease comes from just descriptive research carried out in Africa [14]. Significantly, the lack of comprehensive evidence regarding the efficacy of treatment methods necessitates a thorough examination. The aim of this study is to conduct a thorough analysis of the available data regarding to therapy methods for the treatment of mpox that have been approved and deemed accessible by primary authorities. The focus will be on their application in vulnerable patient populations. Additionally, potential areas for future research will be identified to address any knowledge gaps in this field [15].

## 2 Monkeypox Virus Spreading: Patterns and Epidemiological Insights

The zoonotic characteristics of diseases were recognized early on, but the identification of precise reservoir hosts has proven to be challenging and remains unresolved. Epidemiological investigations were undertaken in order to comprehensively understand the prevalence of diseases, their rates of occurrence, and the contributing elements that facilitate their transmission throughout human populations. During the 1980s, scholars categorized instances into two distinct groups: primary cases, which involved exposure to non‐human animals, and secondary cases, which were only associated with exposure to sick individuals [16].

In 1970, human mpox was documented for the first time in the Democratic Republic of the Congo (DRC) [17]. Subsequently, a total of over 400 cases of mpox were recorded in Africa during the period spanning from 1970 to 1990. The overwhelming majority of these cases were detected in the Democratic Republic of the Congo [18], Following that, a total of six instances were recorded in the Central African Republic (CAR) [19], 2 cases in Cameroon [20], 3 cases in Nigeria [21], 2 cases in Ivory Coast [22], 4 cases in Liberia [23] and 1 case each in Sierra Leone and Gabon [20]. In the year 1990, Cameroon experienced four instances of suspected mpox, with one of these cases being officially verified [24]. Democratic Republic of the Congo encountered an extended occurrence of hMPVX in the year 1996. In February 1996, the confirmation of a patient marked the beginning of the reported instances of mpox [25]. Between the months of February and August in the year 2001, a cumulative count of 16 instances of mpox were detected within the geographical region known as the province of Equateur, situated the dried crusty surface of a healing skin wound or sore [26]. In Democratic Republic of the Congo (DRC), a research investigation carried out by Anne et al. [27] between 2001 and 2004 focused on the analysis of vesicle secretions and the dried crusty surface of a healing skin wound or sore obtained out of 136 individuals who were infer the cause being afflicted with mpox. A cohort of 51 individuals who received a diagnosis of mpox were identified. A total of 51 people diagnosed with mpox were identified.

The global importance of human mpox was of limited significance till the first strongest outmatch occurred outside of Africa, notably in the North America 2003 [5]. A total of forty‐seven instances of mpox were documented, encompassing both confirmed cases (with thirty‐seven patients) and suspected cases (involving ten patients). It was determined that all individuals who contracted the virus had come into contact with pet ground squirrels (*Cynomys spp*.) [5, 28, 29].

Doshi et al. [30] endeavored to do an investigation on the epidemiology of mpox in the Democratic Republic of Congo amongst the outbreak that occurred in 2017 [30]. Beer and Rao's systematic review of hMPXV (human monkeypox virus) outbreaks provides valuable insight into this topic [31]. In this study, the authors have endeavored to illustrate the potential transmission of the virus from animals to humans or from humans to humans, as well as the mortality rate and epidemiological aspects of the disease. Nevertheless, it is imperative to comprehend the epidemiological dynamics of the latest outbreaks of MPX. Kraemer et al. [32] endeavored to depict epidemiological data in real‐time. The authors have presented a record of the aggregated tally of verified cases throughout the current epidemic in the year 2022 [32].

## 3 Transmission Dynamics of Monkeypox

**Figure 1:**
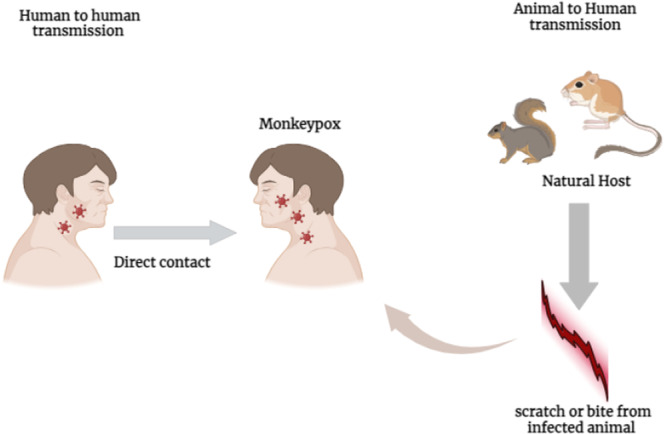
The transmission pathways of the mpox (monkeypox) virus. Mpox is primarily transmitted through direct contact with infected animals (e.g., rodents or non‐human primates) or their body fluids/tissues. Human‐to‐human transmission occurs mainly via close physical contact with an infected person, including contact with skin/mucosal lesions (sores), lesion exudate/crusts, and other body fluids, and through exposure to respiratory secretions during prolonged face‐to‐face interaction. Indirect transmission can occur through contaminated fomites (e.g., bedding, clothing, towels, or surfaces). Airborne transmission is considered uncommon but may occur in exceptional circumstances in healthcare settings during close‐range exposure or certain aerosol‐generating situations.

The primary mode of spread of the Monkeypox virus to humans is through direct contact with several species of wild animals, such as rats and monkeys. However, it is noteworthy that human‐to‐human transmission is also a common occurrence. Additionally, transmission can occur through contact with a skin lesion on an individual who is sick, as shown in Figure 1 [33]. The prevalence of the monkeypox virus has increased significantly following the successful eradication of smallpox, making it the predominant orthopoxvirus [34].

Based on prior accounts, the monkeypox virus was not previously characterized by a high level of contagiousness. Between 1980 and 1984, it was observed that the CB (Congo Basin) clade of mpox in the Democratic Republic of the Congo exhibited a basic reproduction number (R_0_) that was below 1 [35]. As proposed in a study, the reproduction number is basically the average number of secondary cases contracted from an infected population. As an indication to evaluate the contagiousness of infections, R_0_ might represent the biological mechanism of transmission and the rate of contact or interaction among members of the host population [36, 37].

Mpox is documented as an epidemic in Africa, where it is spread primarily through contact with indigenous rodents. Travel‐associated cases of mpox exhibit a restricted capacity for secondary transmission, hence rendering human‐to‐human transmission inefficient [38]. Based on preliminary assessments derived from the examination of the initial cohort of 255 individuals diagnosed with mpox in Italy during the year 2022 using polymerase chain reaction (PCR) testing, it has been determined that the estimated reproduction number for men who engage in sexual activity with other men is 2.43 [39].

In contrast, it is noteworthy that a significant proportion of individuals diagnosed with mpox did not exhibit fresh travel history to the native areas of Africa, including Nigeria, the Democratic Republic of Congo, and various locations in central and western Africa [40]. High rates of transmission between humans during the present outbreak suggest that close contact is the virus's major mechanism of transmission [41, 42].

## 4 Pathogenic Mechanisms of Monkeypox Virus

**Figure 2:**
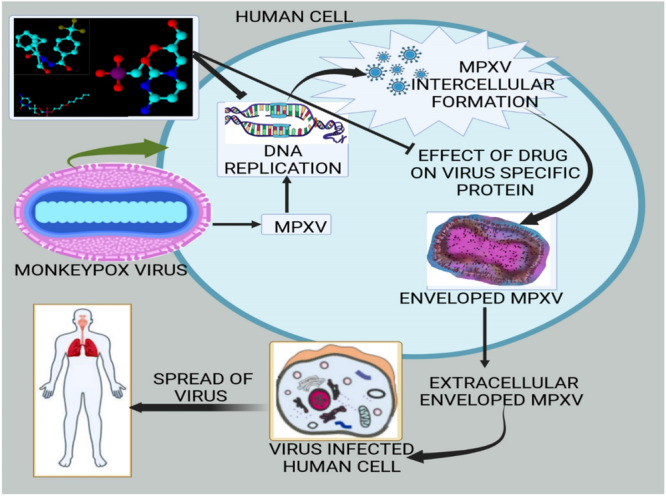
Pathogenesis of MPXV.

The virion forms that are used by MPXV to enter host cells include intracellular mature virus (IMV) and extracellular enveloped virus (EVV), which bind and fuse to host receptors. Having uncoated, viral DNA replicates itself in the cytoplasm in early, intermediate, and late phases, leading to the production of new virions with little host dependence. The virus is carried by immune cells, monocytes and dendritic cells to facilitate the spread and infect the cells. Virus prevents apoptosis through proteins such as P1L and C7L, which block host pro‐apoptotic pathways to extend the life of infected cells. It also suppresses NK cell functioning by binding to NKG2D receptors with OMCP and inhibits interferon responses, which affects the anti‐viral immunity. The adaptive immunity is also weakened by lymphoid depletion in the spleen, tonsils, and nodes. MPXV is tropic to skin (resulting in lesions), lungs (resulting in bronchopneumonia) and gastrointestinal tract (with lesions in GALT‐loaded regions such as colon). The granulocytes, monocytes and lymphocytes are blood cells that assist in replication and viremia in animal models such as nonhuman primates [43]. The virion forms that are used by MPXV to enter host cells include intracellular mature virus (IMV) and extracellular enveloped virus (EVV), which bind and fuse to host receptors, leading to viral replication and dissemination within host tissues, as shown in Figure 2.

The process of viral DNA replication, transcription, and virion packaging necessitates the involvement of several proteins [44]. The MPXV exhibits the ability to enter the host cell through fusion and macropinocytosis mechanisms [45]. The presence of Internal Mature Virus (IMV) and External Enveloped Virus (EEV) introduces complications to the entry and release mechanisms due to their distinct lipid membranes and surface proteins, rendering them non‐identical to each other [46] as explained in Figure 2. Moreover, it is worth noting that the Congo clade of the Monkeypox virus (MPXV) may exhibit distinct characteristics compared to the West African clade. Specifically, the Congo clade has been observed to display higher levels of virulence and pathogenicity [40].

Subsequently, the virus proceeds to invade the respiratory and oral mucosa, primarily targeting the epithelial cells that line the upper, middle, and lower airways. Currently, in the progression of the infection, the patient exhibits no discernible symptoms, and there are no visible lesions present in the oropharynx [44]. The pathogenic mechanism of MPXV described by [45] Sharma et al. (2022). The process of viral replication is facilitated by the existence of resident immune cells in the adjacent tissue, encompassing a diverse range of antigen‐presenting cells such as monocytes, macrophages, B cells, and dendritic cells. The phenomenon of orthopoxvirus migration from the site of infection to the draining lymph nodes is currently a subject of active scientific discourse. Evidence suggests that mouse dendritic cells infected with VACV exhibit migration from the lung epithelium to the draining lymph nodes, potentially contributing to the dissemination of the virus. Previous studies have demonstrated that the infection of human monocyte‐derived dendritic cells with VACV is ineffective due to its ability to hinder dendritic cell maturation and migration [44, 45].

The findings presented in this study provide evidence that contradicts the concept suggesting that dendritic cells play a role in the initial dissemination of vaccinia virus (VACV) through the lymphatic system. VACV spreads quickly in the lymphatic system, causing it to migrate to nearby lymph nodes upon inoculation [40].

From January 1 to July 7, 2022, [46] performed a cross‐sectional investigation across Europe. The study indicated the rapid spread of human mpox across the four subregions, affecting 30 European nations and 6077 people. Mpox cases also increased, with incidences documented in Western Europe (2599 cases, accounting for 42.76% of the total), Southern Europe (1932 cases, representing 31.79% of the total), Northern Europe (1487 cases, comprising 24.46% of the total), and Eastern Europe (59 cases, accounting for 0.97% of the total) [46]. Mpox consequences may include encephalitis, pneumonia, and subsequent bacterial infections. When the skin lesions become secondary infected, secondary bacterial infections, such as staphylococcal or streptococcal infections, might ensue [47, 48]. The aforementioned discovery was supported by a study that utilized a cynomolgus macaque model of aerosolized MPXV infection. In this study, it was seen that viral replication was notably present in the tonsils, as well as the mandibular and mediastinal lymph nodes, during the early phases of infection [49, 50].

## 5 Genomic Organization of Monkeypox Virus (MPXV)

The MPXV genome is ~197 kb long and contains a conserved central coding region ( ~ 101,476 bp) encoding essential replication and transcription genes. This region is flanked at both ends by 6379 bp terminal inverted repeats (ITRs) that include an ~80 bp hairpin, 54 bp tandem repeats, and NR1/NR2 regions, which mainly encode virulence and host‐interaction factors.

## 6 Clinical Manifestations

MPVX generally induces a transient cutaneous eruption in non‐human primates. The initial clinical manifestations of the disease consist of papules on the epidermis, ranging in size from 1 to 4 mm [51], which subsequently progress into pustules and eventually form crusts. A characteristic feature of a lesion is the presence of a center that exhibits a red coloration, necrotic tissue, and a depressed appearance, which is encompassed by an excessive growth of the epidermis. After the administration of vaccinations, instances of MPVX‐induced maladies were identified in diverse rodent species, encompassing prairie dogs, dormice, and squirrels [52].

**Figure 3:**
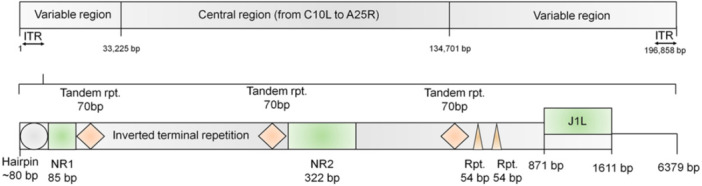
Monkeypox virus (MPXV) genomic organization.

The clinical manifestations observed in each of the patients depicted in Figure 3 exhibit variability. Nevertheless, typical manifestations such as elevated body temperature, loss of body mass, discharge from the nasal cavity, bouts of coughing, respiratory impairment, and the presence of oral lesions may serve as potential indicators of the aforementioned ailment [53]. Nevertheless, typical manifestations, such as elevated body temperature, loss of body mass, discharge from the nasal passages, bouts of coughing, respiratory impairment, and the presence of oral lesions, may serve as potential indicators of the ailment [52].

The current outbreak has observed that MSM individuals constitute a significant proportion of reported cases, frequently presenting with genital lesions or a vesicular‐pustular rash [53]. The observation that the perineum and vaginal area frequently exhibit rash symptoms implies that the illness may have been acquired through sexual intercourse. Mpox is frequently misidentified as other sexually transmitted illnesses (STIs), including granuloma inguinale, molluscum contagiosum, chancre, or herpes simplex virus [54].

However, the clinical signs of mpox infection have a resemblance to a less severe presentation of smallpox. The differentiation lies in the fact that lymphadenopathy is caused by infection with MPXV, as opposed to smallpox. The initial indications of an MPXV infection encompass fever, chills, headache, muscular pains, backaches, and lethargy, which afterward progress to a state of exhaustion [55]. Mpox infection is a viral disease caused by the monkeypox virus. It is a zoonotic disease. The incubation period for MPVX is commonly observed to range from seven to 14 days, while in some cases it may extend up to 21 days. Following the initiation of pyrexia and the manifestation of a facial rash, the pathogenic agent disseminates to additional anatomical regions within the body [56]. Lesions typically originate in the oropharynx before disseminating throughout the organism. Serum antibodies can be identified around 2 weeks following exposure. The mortality rate of the pathogenic viral strain of mpox can range from 1% to 10%, depending on its genealogy and the availability of modern healthcare [55].

## 7 Differential Diagnosis of Mpox From Other Infections

Early detection of mpox is critical for effective management and containment of its spread. Deep learning, a subfield of machine learning, uses convolutional neural networks to analyze and learn from large datasets. This method has greatly aided in the identification of diseases like mpox. Deep learning algorithms can analyze large amounts of data from medical imaging, lab tests, and patient records, leading to more accurate illness detection [57]. There are considerable difficulties in the differential diagnosis of mpox due to the overlapping clinical presentations of mpox with many sexually transmitted infections (STIs) and other vesicular diseases, which have been important since many STIs have signs and symptoms overlapping with mpox [58].

More than 40% of mpox cases involved co‐infection with sexually transmitted infections, including Chlamydia trachomatis, Neisseria gonorrhoeae, Treponema pallidum, and herpes simplex virus during the outbreaks of 2022–2023 [59]. The main differential diagnostic problems with mpox include possible similarities to smallpox, varicella, measles, bacterial skin infections, drug allergies, and syphilis [60]. Diagnosis of pustular lesions differential includes mpox, herpes simplex virus, molluscum contagiosum, cutaneous cryptococcosis, cutaneous cytomegalovirus, syphilis, and lymphogranuloma venereum. Lymph node enlargement was considered a distinguishing feature by Chen et al., who diagnosed Sackheim's disease (cutaneous cryptococcosis) based on biopsies [61].

However, a problem with the current types of diagnostic real‐time PCR in use at CLIA laboratories and reference gene banks (such as Mayo Clinic and ARUP Laboratories) in the United States during the 2022–2023 outbreaks, namely that they are all Pan‐OPXV real‐time PCR, cannot differentiate MPXV from other OPXVs, e.g., smallpox vaccine strains or molluscum contagiosum virus (MOCV) [62, 63].

## 8 Sample Collection Protocols

Examples that are appropriate for the detection of mpox include surface and/or skin samples, such as exudate swabs and lesion crusts [64]. To collect an adequate amount of viral DNA, it is recommended to vigorously rub the swab against the lesion (Table 1). It is advisable to collect samples from multiple locations and various lesions, if feasible. These swab samples should be delivered to the laboratory either in a dry medium or a Viral Transport Medium (VTM). It is permissible to arrange two lesions of the same type within a single tube. However, it is advised to avoid combining lesions, crusts, and vesicular fluids within the same tube during transportation. In the process of doing an MPX diagnostic, there exists a potential for encountering either a false negative outcome, whereby the test erroneously indicates the absence of mpox despite its presence, or a false positive outcome, wherein the test inaccurately suggests the presence of mpox despite its absence [66].

**Table 1:** HMPX Diagnostic Testing [65].

Sr. No	Diagnostic assessment	Sample source	Outcomes	Interpretations
Non‐specific tests				
1	White Blood Cell Count	Blood	Elevated, nearing or surpassing 4.0 × 10 ^ 3–11.0 × 10 ^ 3/μL high range	Suggestive of possible infection
2	Alanine Aminotransferase (ALT)	Blood	Below or close to the 12–78 U/L lower threshold	Indicative of potential infection
3	Aspartate Aminotransferase (AST)	Blood	Below or proximate to the 15–37 U/L lower limit	Pointing to potential infection
Specific tests				
1	NAAT (Nucleic Acid Amplification Test)	Lesion Fluid	Utilizes Nucleic Acid Amplification to detect MPVX DNA	Detection of MPVX DNA signifies positivity
2	Anti‐Orthopoxvirus IgG and IgM Analysis	Blood	Assessments for exposure to Orthopoxvirus	Detection of positive antibodies indicates exposure
3	Immunohistochemical Examination	Blood	Investigation for orthopoxvirus‐specific antigens	Detection of antigen implies positive finding
4	Electron Microscopy Analysis	Biopsy Sample, Scab Scrapings, Vesicular Secretions	Morphological identification of poxviruses using electron microscope	Positive observation reveals brick‐shaped organisms, sized around 200–300 nm
5	Viral Culture Technique	Lesion Fluid	Specimen cultured from patient sample and propagated in appropriate media	Positive growth indicates viral presence

### 8.1 Testing Methods for Monkeypox Virus

#### 8.1.1 Molecular Tests

Molecular assays, which are alternatively referred to as nucleic acid amplification tests, are employed to identify certain genes associated with the monkeypox virus. These tests serve as the definitive means of confirming the presence of mpox. The utilization of Orthopoxvirus PCR, which focuses on genes shared by several orthopoxviruses such as smallpox and vaccinia, is limited to serving as a preliminary diagnostic tool rather than a definitive diagnostic test [67].

The inclusion of additional infectious diseases in the diagnostic process can be advantageous in distinguishing mpox from other similar conditions [68]. Nevertheless, the coexistence of various pathogens within skin lesions does not always negate the possibility of the acquisition of the monkeypox virus by an individual. Numerous instances of co‐infection involving the monkeypox virus alongside other pathogenic agents have been documented [69].

#### 8.1.2 Virus Isolation

Although viral isolation can be used as a confirmatory test, it has limited use in medical institutions due to its complex method and low sensitivity. Isolating viruses requires BSL‐3 or higher methods and facilities [70].

#### 8.1.3 Serology Parameters

Mpox antibody testing typically targets antibodies that respond to orthopoxviruses other than the monkeypox virus. As a result, these tests are not appropriate for serving as confirming diagnostic tools [71]. However, if the skin lesions have healed completely, antibody testing may be considered useful for performing sero‐surveillance studies and diagnosing suspicious cases. The antibody test for orthopoxvirus has shown cross‐reactivity among those who have received the smallpox vaccine [72].

#### 8.1.4 Indications for Molecular Tests

Molecular diagnostic assays are used to validate suspected mpox cases and to decide whether patients with confirmed mpox infections should be discharged from medical facilities. Furthermore, it is important to examine persons who have had personal contact with confirmed cases, even if they do not exhibit symptoms [67].

#### 8.1.5 Requirements for Molecular Tests

Real‐time polymerase chain reaction (PCR) is the most often used molecular diagnostic tool for mpox. Conventional PCR, on the other hand, can be a feasible alternative for this purpose [73]. As confirmatory tests, clade‐specific PCR, which is intended to distinguish between several clades of mpox, and multiplex PCR, which simultaneously identifies other diseases, can both be used [74].

#### 8.1.6 MpoxmpoxPCRs With Non‐VARV OPXV (NVO) and Clade Specific PCR

In the first place, real‐time PCRs can also be carried out using non‐VARV OPXVs and Clade II‐specific MPXVs as templates [59, 75]. The Centers for Disease Control and Prevention in the United States does so, as do some private laboratory partners, which provide tests out of their facilities; all of these tests have proved invaluable in managing the 2022–2023 subclade IIb mpox outbreak. However, the NVO PCR cannot differentiate between subclade 1b and Clade 2 MPXVs. The NVO PCR has, however, no discrimination between subclade Ib and clade II MPXV. To address the 2024 subclade Ib mpox PHEIC, a modified algorithm to interpret mpox real‐time PCRs with NVO and II MPXV PCR targets [76], should be modified to accommodate a scenario of subclade Ib MPXV. In particular, productively positive NVO targets together with a negative clade II‐specific MPXV target should be presumptively diagnosed or suspected of clade I MPXV [76].

Another important thing to manage mpox outbreaks in Central Africa, the WHO has included Cobas MPXV assay (Roche Molecular, Pleasanton, CA, USA) and (Abbott Molecular, Des Plaines, IL, USA) in Emergency Use Listing (EUL). Both the assays are applicable in a centralized reference laboratory where trained clinical lab staff with expertise in both PCR methodology and in vitro diagnostic methodology are found. Nonetheless, these mpox tests of high throughput are not able to distinguish between the clades I and II MPXV. The cobas MPXV assay and Alinity m MPXV assay target MPXV genes F3L and B21R/B22R, and MPXV genes B7R and J2R, respectively, both of which do not distinguish between clades I and II MPXV [75].

Moreover, no MPXV antigen rapid diagnostic tests (RDTs) are available, which would allow diagnosing the acute infection quickly by identifying viral proteins at the POC. Antigen‐based RDTs are less sensitive than nucleic acid amplification tests (NAATs), and in some instances, there is no ability to exclude infection with a negative antigen test. No antigen RDTs have met the minimum sensitivity requirement on mpox testing in a recent systematic search by the Africa CDC Diagnostic Advisory Committee [76].

## 9 Measures for Preventing Mpox

The immune system's reactions to a specific orthopoxvirus can exhibit cross‐reactivity with other orthopoxviruses, leading to differential degrees of protection contingent upon the level of genetic relatedness between the distinct orthopoxvirus strains [77]. There exists a notion suggesting that the rise in mpox cases subsequent to the discontinuation of smallpox immunization can be attributed to a progressively immunologically inexperienced population.

The phenomenon of immunological cross‐reactivity has facilitated the development of diverse animal models for studying smallpox infection, which have been crucial in evaluating the efficacy of vaccinations and antiviral treatments [78]. The primary cause of this cross‐reactivity can be attributed to two main causes. The significant level of sequence similarity observed among orthopoxviruses, particularly in immunologically significant proteins, results in a considerable quantity of common immune epitopes [79].

Second, the reaction is very comprehensive, covering at least 24 different membrane and structural proteins [80]. T‐cell responses are capable of identifying epitopes across a diverse array of viral proteins. CD4 T cells exhibit a preference for structural proteins, while CD8 T cells primarily target proteins that are produced in the early stages of infection, such as virulence factors. The presence of a neutralizing antibody has been linked to protection against variola virus‐caused smallpox in humans [81] and against other orthopoxviruses in animal models. Although T lymphocytes are not necessary to offer protection, they do help clear the body of viruses [82].

Based on the existing evidence, it may be inferred that prior administration of the smallpox vaccine may provide an immunity boost towards the monkeypox virus and potentially amplify the clinical manifestation of the infection [24]. At present, the US Strategic National Stockpile (SNS) comprises three smallpox vaccines: JYNNEOSTM (also referred to as IMVAMUNE, IMVANEX, MVA‐BN) and ACAM2000®, which hold licenses for smallpox vaccination, while the Aventis Pasteur Smallpox Vaccine (APSV) may be employed for smallpox purposes through an investigational new drug (IND) protocol.

JYNNEOSTM is a live viral vaccine that is developed from the MVA‐BN strain of modified vaccinia Ankara‐Bavarian Nordic. This particular strain is an attenuated orthopoxvirus that exhibits a lack of replication capability [83]. In September 2019, the United States Food and Drug Administration (FDA) granted licensure to the aforementioned product. Presently, it is specifically recommended for employment in the prevention of smallpox and mpox afflictions among individuals aged 18 years or older who have been identified as being at a heightened susceptibility to contracting smallpox or mpox infections mpox [84]. The IMVANEX® vaccine has received approval in Europe for the prevention of smallpox. However, it is worth noting that in the United Kingdom, in response to cases of mpox, this immunization has been used off‐label [85].

ACAM2000®, in addition, comprises live vaccinia virus. The Food and Drug Administration (FDA) granted licensure to the vaccine in August (2007), effectively displacing the prior Imvamune known as Dryvax® that had been pull back its maker [86]. ACAM2000® is specifically intended for the active immunization of individuals identified as being at a heightened risk of contracting smallpox illness. The Centers for the prevention and control of disease (CDC) has been established an urgent situation access Investigated the New Drug (IND) procedure, which permits the utilization of ACAM2000® for the management of orthopoxvirus infections other than variola, like mpox, in the event of an outbreak [87].

ACAM2000® was primarily created to actively immunize individuals who have been recognized as having a higher risk of contracting smallpox disease. When an outbreath occurs, the Center for the Prevention and Disease Control (CDC) has implemented an urgent access Investigation of New Drug (IND) protocol, which allows ACAM2000® to be used for the treatment of non‐variola orthopoxvirus infections, such as mpox [88]. A brief comparison between ACAM2000® and JYNNEOS™ vaccine is explained in Table 2 below.

**Table 2:** Distinguish Between the Vaccines Available for HMPX [89].

	ACAM2000®	JYNNEOS™
Type of Vaccine	Live cowpox Vaccinia virus immunization	Replication‐deficient live virus immunogen
Time to Elicit Immune Response	Evident 28 days following vaccination	Manifests 14 days post the second dose
Dosage Regimen	Single dose	Administered in 2 doses separated by 28 days
Notable Adverse Effects	Possibility of inadvertent vaccinia virus inoculation, myopericarditis, and prolonged wound healing at the injection site	Injection site reactions leading to muscle discomfort, headaches, and fatigue
Contraindications During Pregnancy	Not recommended during pregnancy	Deemed safe during pregnancy or breastfeeding based on animal studies
General Restrictions	Inadvisable for individuals with cardiovascular ailments, congenital or acquired autoimmune disorders like HIV, particular skin conditions, or those using topical steroids for ocular ailments	
Approval Status	Specifically approved for smallpox prevention	Authorized in 2019 for preventing smallpox and monkeypox

## 10 Therapeutic Interventions of Monkeypox Disease

### 10.1 Supportive Care

The majority of individuals afflicted with mpox infection experience spontaneous recovery in the absence of medical intervention. People who are having gastrointestinal symptoms like vomiting and diarrhea will need to be rehydrated either orally or intravenously in order to prevent further loss of fluids from the gastrointestinal system [90].

### 10.2 Antivirals

There are a number of antiviral medications that have shown potential efficacy in the treatment of mpox infections. It is important to remember that these medications were first approved for the treatment of smallpox, mostly due to their positive effects in animal models. This was the primary justification for the approval of these treatments. Despite the fact that there have been trials conducted on human subjects to evaluate the effects of these drugs, the efficacy of these treatments has not been completely characterized [91].

a. **Tecovirimat**
  Tecovirimat, known by its commercial name TPOXX and code name ST‐246, is a pharmaceutical agent classified as an antiviral medication [92]. As previously stated, tecovirimat received initial approval from the FDA in 2018 for the purpose of treating smallpox. However, the European Medicines Agency has just approved it for the treatment of mpox sickness. The initial discovery of this antiviral medication occurred in 2002 using a high‐throughput screening process. Subsequent studies have provided evidence of its efficacy against many orthopoxviruses, such as cowpox, rabbitpox, ectromelia, vaccinia virus, as well as variola and MPXV [93].
  Tecovirimat exerts its pharmacological effect through the inhibition of the VP37 protein. It is widely thought that all species belonging to the orthopoxvirus genus possess the ability to encode this particular protein. The prevention of the interaction between VP37 and GTPase and TIP47 by the inhibition of this protein will result in the obstruction of the formation of essential enveloped virions [94]. Preclinical studies have shown tecovirimat to be beneficial against MPXV; these include seven animal studies and one study performed in a laboratory environment. According to the findings of the animal experiments, tecovirimat exhibited a significant reduction in mortality among animals infected with MPXV, resulting in a survival rate of at least 90% [95]. Nevertheless, the effectiveness in reducing mortality was observed to decline in rats that received treatment after a delay following exposure [96].
b. **Brincidofovir and Cidofovir**
  Treatment of MPXV infection is limited, and antiviral drugs licensed and approved against human smallpox disease should be used, such as tecovirimat (commercially branded as TPOXX®) [97], and brincidofovir (BCV commercially branded as Tembexa®), or its bioactive constituent, cidofovir (CDV commercially branded as Vistide®). Tecovirimat is an integrase inhibitor of the OPXV F13 envelope protein (also referred to as VP37) [98]. Brincidofovir is an antiviral medication (orally formulated) targeting smallpox and various other DNA viruses, which is approved to treat infection with smallpox virus (variola) in humans and has been granted emergency use authorization to treat mpox infection [99].

CDV is a nucleotide analog that is a broad‐spectrum viral DNA polymerase inhibitor, and BCV is a lipid conjugate of CDV with a better oral bioavailability and safety profile [100, 101]. As a result, alternative treatment interventions are needed, and several case studies have reported effective clinical response when tecovirimat‐resistant infections were later treated with the CDV or BCV [102, 103].

## 11 Conclusion

In this narrative review, authors highlight the paradigm shift in which the monkeypox virus (MPXV) has transformed into an unnoticed (in Central and Western Africa) but dangerous zoonotic infection to an important global health alert. The resurgence of 2022–2023 in the non‐endemic world and in interconnected sexual networks resulted in the World Health Organization putting it as a developing threat with moderate public health concern. This change emphasizes the great need to be more vigilant on the world level and to adopt strict infection control measures.

Prevention measures are still of first priority, especially the tactical application of vaccines like JYNNEOS and ACAM2000®. These are cross‐protective as the orthopoxviruses are highly similar in their sequence. In the context of clinical management, a lack of specialized antiviral treatments has become a significant weakness in the modern medical range of treatment. Although investigational medications, such as cidofovir and brincidofovir, demonstrate their potential in the laboratory and animal models, supportive care, which is aimed at reducing symptoms and preserving fluid balance, remains the fundamental part of treating infected individuals.

Finally, the consequences of the mpox revival on the global population health highlight the importance of maintaining an interest in the research in order to fill the gaps in therapeutic knowledge and to make sure that modern healthcare is accessible both in the endemic and non‐endemic areas.

## Author Contributions

**Muslim Bin Aqeel:** conceptualization, validation, methodology, writing – review and editing, writing – original draft, visualization, data curation. **Asif Hanif:** conceptualization, data curation, writing – review and editing, writing – original draft. **Shahzad Ahmad:** data curation, writing – review and editing, writing – original draft. **Fatima Iftikhar Shah:** data curation, writing – review and editing, writing – original draft. **Benish Javed:** data curation, writing – review and editing, writing – original draft. **Shazia Iqbal:** data curation, writing – review and editing, writing – original draft.

## Funding

The authors have nothing to report.

## Conflicts of Interest

The authors declare no conflicts of interest.

## Transparency Statement

The lead author Muslim Bin Aqeel, Shahzad Ahmad affirms that this manuscript is an honest, accurate, and transparent account of the study being reported; that no important aspects of the study have been omitted; and that any discrepancies from the study as planned (and, if relevant, registered) have been explained.

## Data Availability

The authors have nothing to report.

## References

[1] Z. Yang , “Monkeypox: A Potential Global Threat?,” Journal of Medical Virology 94, no. 9 (2022): 4034–4036. 35614026 10.1002/jmv.27884 PMC9283296

[2] J. G. Rizk , G. Lippi , B. M. Henry , D. N. Forthal , and Y. Rizk , “Prevention and Treatment of Monkeypox,” Drugs 82, no. 9 (2022): 957–963. 35763248 10.1007/s40265-022-01742-y PMC9244487

[3] O. Mitjà , D. Ogoina , B. K. Titanji , et al., “Monkeypox,” Lancet 401, no. 10370 (2023): 60–74. 36403582 10.1016/S0140-6736(22)02075-X PMC9671644

[4] A. Gessain , E. Nakoune , and Y. Yazdanpanah , “Monkeypox,” New England Journal of Medicine 387, no. 19 (2022): 1783–1793. 36286263 10.1056/NEJMra2208860

[5] K. D. Reed , J. W. Melski , M. B. Graham , et al., “The Detection of Monkeypox in Humans in the Western Hemisphere,” New England Journal of Medicine 350, no. 4 (2004): 342–350. 14736926 10.1056/NEJMoa032299

[6] Outbreak, M.‐C.M ., Situation Update (World Health Organization. Available online, 2022). (accessed on 17 June 2022), https://www.who.int/emergencies/disease-outbreak-news/item/2022-DON393.

[7] R. Sah , A. Mohanty , A. Reda , et al., “Monkeypox: A Potential Pandemic at Door of Asia,” Annals of Medicine & Surgery 81 (2022), https://journals.lww.com/annals-of-medicine-and-surgery/fulltext/2022/09000/monkeypox__a_potential_pandemic_at_door_of_asia.177.aspx. 10.1016/j.amsu.2022.104509 PMC9464850 36106066

[8] J. P. Thornhill , S. Barkati , S. Walmsley , et al., “Monkeypox Virus Infection in Humans Across 16 Countries—April–June 2022,” New England Journal of Medicine 387, no. 8 (2022): 679–691. 35866746 10.1056/NEJMoa2207323

[9] S. L. Haller , C. Peng , G. McFadden , and S. Rothenburg , “Poxviruses and the Evolution of Host Range and Virulence,” Infection, Genetics and Evolution 21 (2014): 15–40. 10.1016/j.meegid.2013.10.014 PMC3945082 24161410

[10] A. L. Hughes , S. Irausquin , and R. Friedman , “The Evolutionary Biology of Poxviruses,” Infection, Genetics and Evolution 10, no. 1 (2010): 50–59. 10.1016/j.meegid.2009.10.001 PMC2818276 19833230

[11] J. Kaler , A. Hussain , G. Flores , S. Kheiri , and D. Desrosiers , “Monkeypox: A Comprehensive Review of Transmission, Pathogenesis, and Manifestation,” Cureus 14, no. 7 (2022): e26531, 10.7759/cureus.26531. 35928395 PMC9345383

[12] G. Guarducci , B. R. Porchia , C. Lorenzini , and N. Nante , “Overview of Case Definitions and Contact Tracing Indications in the 2022 Monkeypox Outbreak,” Le Infezioni in Medicina 31, no. 1 (2022): 13–19. 36908385 10.53854/liim-3101-3 PMC9994831

[13] R. A. Farahat , R. Sah , A. A. El‐Sakka , et al., “Human Monkeypox Disease (MPX),” Le Infezioni in Medicina 30, no. 3 (2022): 372–391. 36148174 10.53854/liim-3003-6 PMC9448318

[14] F.‐M. Lum , A. Torres‐Ruesta , M. Z. Tay , et al., “Monkeypox: Disease Epidemiology, Host Immunity and Clinical Interventions,” Nature Reviews Immunology 22, no. 10 (2022): 597–613. 10.1038/s41577-022-00775-4 PMC9443635 36064780

[15] D. T. Ivanov , Y. A. Slabakova , R. M. Argirova , and T. K. Valkov , “Antivirals for the Treatment of Monkeypox: Utilization in the General and HIV‐Positive Population and Gaps for Research. A Short Narrative Review,” Le Infezioni in Medicina 31, no. 2 (2023): 186–194. 37283638 10.53854/liim-3102-6 PMC10241405

[16] I. K. Damon , “Status of Human Monkeypox: Clinical Disease, Epidemiology and Research,” Vaccine 29 (2011): D54–D59. 22185831 10.1016/j.vaccine.2011.04.014

[17] I. D. Ladnyj , P. Ziegler , and E. Kima , “A Human Infection Caused by Monkeypox Virus in Basankusu Territory, Democratic Republic of the Congo,” Bulletin of the World Health Organization 46, no. 5 (1972): 593–597. 4340218 PMC2480792

[18] Z. Jezek , B. Grab , K. M. Paluku , and M. V. Szczeniowski , “Human Monkeypox: Disease Pattern, Incidence and Attack Rates in a Rural Area of Northern Zaire,” Tropical and Geographical Medicine 40, no. 2 (1988): 73–83. 2841783

[19] L. Khodakevich , R. Widy‐Wirski , I. Arita , S. S. Marennikova , J. Nakano , and D. Meunier , “Monkey Pox Virus Infection in Humans in the Central African Republic,” Bulletin de la Societe de Pathologie Exotique et de ses Filiales 78, no. 3 (1985): 311–320. 2992830

[20] A. Meyer , J. J. Esposito , F. Gras , T. Kolakowski , M. Fatras , and G. Muller , “First Appearance of Monkey Pox in Human Beings in Gabon,” Medecine Tropicale: Revue du Corps de Sante Colonial 51, no. 1 (1991): 53–57. 1649373

[21] O. Faye , C. B. Pratt , M. Faye , et al., “Genomic Characterisation of Human Monkeypox Virus in Nigeria,” Lancet Infectious Diseases 18, no. 3 (2018): 246. 10.1016/S1473-3099(18)30043-4 PMC9628790 29361427

[22] F. Merouze and J. J. Lesoin , “Monkeypox: Second Human Case Observed in Ivory Coast (Rural Health Sector of Daloa,” Medecine Tropicale: Revue du Corps de Sante Colonial 43, no. 2 (1983): 145–147. 6306382

[23] B. Lourie , P. G. Bingham , H. H. Evans , S. O. Foster , J. H. Nakano , and K. L. Herrmann , “Human Infection With Monkeypox Virus: Laboratory Investigation of Six Cases in West Africa,” Bulletin of the World Health Organization 46, no. 5 (1972): 633–639. 4340223 PMC2480791

[24] D. L. Heymann , M. Szczeniowski , and K. Esteves , “Re‐Emergence of Monkeypox in Africa: A Review of the Past Six Years,” British Medical Bulletin 54, no. 3 (1998): 693–702. 10326294 10.1093/oxfordjournals.bmb.a011720

[25] V. Mukinda , G. Mwema , M. Kilundu , D. Heymann , A. Khan , and J. Esposito , “Re‐Emergence of Human Monkeypox in Zaire in 1996,” Lancet 349, no. 9063 (1997): 1449–1450. 9164323 10.1016/S0140-6736(05)63725-7 PMC9533927

[26] H. Meyer , M. Perrichot , M. Stemmler , et al., “Outbreaks of Disease Suspected of Being Due to Human Monkeypox Virus Infection in the Democratic Republic of Congo in 2001,” Journal of Clinical Microbiology 40, no. 8 (2002): 2919–2921. 12149352 10.1128/JCM.40.8.2919-2921.2002 PMC120683

[27] A. W. Rimoin , N. Kisalu , B. Kebela‐Ilunga , et al., “Endemic Human Monkeypox, Democratic Republic of Congo, 2001–2004,” Emerging Infectious Diseases 13, no. 6 (2007): 934–937. 17553242 10.3201/eid1306.061540 PMC2792850

[28] M. G. Reynolds , K. L. Yorita , M. J. Kuehnert , et al., “Clinical Manifestations of Human Monkeypox Influenced by Route of Infection,” Journal of Infectious Diseases 194, no. 6 (2006): 773–780. 16941343 10.1086/505880

[29] Y. Huang , L. Mu , and W. Wang , “Monkeypox: Epidemiology, Pathogenesis, Treatment and Prevention,” Signal Transduction and Targeted Therapy 7, no. 1 (2022): 373. 36319633 10.1038/s41392-022-01215-4 PMC9626568

[30] R. H. Doshi , S. A. J. Guagliardo , J. B. Doty , et al., “Epidemiologic and Ecologic Investigations of Monkeypox, Likouala Department, Republic of the Congo, 2017,” Emerging Infectious Diseases 25, no. 2 (2019): 281–289. 30666937 10.3201/eid2502.181222 PMC6346463

[31] E. M. Beer and V. B. Rao , “A Systematic Review of the Epidemiology of Human Monkeypox Outbreaks and Implications for Outbreak Strategy,” PLoS Neglected Tropical Diseases 13, no. 10 (2019): e0007791. 31618206 10.1371/journal.pntd.0007791 PMC6816577

[32] M. U. G. Kraemer , H. Tegally , D. M. Pigott , et al., “Tracking the 2022 Monkeypox Outbreak With Epidemiological Data in Real‐Time,” Lancet Infectious Diseases 22, no. 7 (2022): 941–942. 35690074 10.1016/S1473-3099(22)00359-0 PMC9629664

[33] E. Alakunle , U. Moens , G. Nchinda , and M. I. Okeke , “Monkeypox Virus in Nigeria: Infection Biology, Epidemiology, and Evolution,” Viruses 12, no. 11 (2020): 1257. 33167496 10.3390/v12111257 PMC7694534

[34] A. Kantele , K. Chickering , O. Vapalahti , and A. W. Rimoin , “Emerging Diseases—The Monkeypox Epidemic in the Democratic Republic of the Congo,” Clinical Microbiology and Infection 22, no. 8 (2016): 658–659. 27404372 10.1016/j.cmi.2016.07.004 PMC9533887

[35] R. Grant , L.‐B. L. Nguyen , and R. Breban , “Modelling Human‐To‐Human Transmission of Monkeypox,” Bulletin of the World Health Organization 98, no. 9 (2020): 638–640. 33012864 10.2471/BLT.19.242347 PMC7463189

[36] G. MacDonald , The Epidemiology and Control of Malaria (Oxford Univ. Pr, 1957, Oxford).

[37] P. E. M. Fine , “Herd Immunity: History, Theory, Practice,” Epidemiologic Reviews 15, no. 2 (1993): 265–302. 8174658 10.1093/oxfordjournals.epirev.a036121

[38] E. M. Bunge , B. Hoet , L. Chen , et al., “The Changing Epidemiology of Human Monkeypox—A Potential Threat? A Systematic Review,” PLoS Neglected Tropical Diseases 16, no. 2 (2022): e0010141. 35148313 10.1371/journal.pntd.0010141 PMC8870502

[39] G. Guzzetta , A. Mammone , F. Ferraro , et al., “Early Estimates of Monkeypox Incubation Period, Generation Time, and Reproduction Number, Italy, May–June 2022,” Emerging Infectious Diseases 28, no. 10 (2022): 2078–2081. 35994726 10.3201/eid2810.221126 PMC9514338

[40] T. P. Velavan and C. G. Meyer , “Monkeypox 2022 Outbreak: An Update,” Tropical Medicine & International Health 27, no. 7 (2022): 604–605. 35633308 10.1111/tmi.13785

[41] H. Kluge and A. Ammon , “Monkeypox in Europe and Beyond–Tackling a Neglected Disease Together,” Eurosurveillance 27, no. 24 (2022): 2200482. 35713025 10.2807/1560-7917.ES.2022.27.24.2200482 PMC9205161

[42] N. Derhab , “Human Monkeypox Virus: A Systematic Critical Review During the Pandemic Peak,” Indian Journal of Medical Microbiology 51 (2024): 100704. 39134221 10.1016/j.ijmmb.2024.100704

[43] A.‐L. Rupp , J. P. Meister , J. Schulze Zur Wiesch , and T. L. Meister , “From Skin Lesions to Multi‐Organ Involvement: Organ Tropism and Pathogenesis of Mpox Virus,” iScience 28, no. 12 (2025): 114209. 41476947 10.1016/j.isci.2025.114209 PMC12752768

[44] S. Myint and D. Taylor‐Robinson , Viral and Other Infections of the Human Respiratory Tract (Springer Science & Business Media, 2012).

[45] V. Sharma , D. Aggarwal , A. K. Sharma , et al., “An Overview on Monkeypox, Current Paradigms and Advances in Its Vaccination, Treatment and Clinical Management: Trends, Scope, Promise and Challenges,” Journal of Pure and Applied Microbiology 16, no. suppl 1 (2022): 3000–3012.

[46] S. A. Meo , A. H. Alsomali , A. A. Almushawah , and A. S. Meo , “Epidemiological Trends of Human Monkeypox Cases in Northern, Southern, Western, and Eastern Regions in Europe: A Cross‐Sectional Study,” Journal of Tropical Medicine 2022, no. 1 (2022): 1–7. 10.1155/2022/4042962 PMC9473903 36118592

[47] J. G. Breman , Kalisa‐Ruti , M. V. Steniowski , E. Zanotto , A. I. Gromyko , and I. Arita , “Human Monkeypox, 1970‐79,” Bulletin of the World Health Organization 58, no. 2 (1980): 165–182. 6249508 PMC2395797

[48] H. Song , N. Josleyn , K. Janosko , et al., “Monkeypox Virus Infection of Rhesus Macaques Induces Massive Expansion of Natural Killer Cells but Suppresses Natural Killer Cell Functions,” PLoS ONE 8, no. 10 (2013): e77804. 24147080 10.1371/journal.pone.0077804 PMC3798392

[49] G. M. Zaucha , P. B. Jahrling , T. W. Geisbert , J. R. Swearengen , and L. Hensley , “The Pathology of Experimental Aerosolized Monkeypox Virus Infection in Cynomolgus Monkeys (Macaca Fascicularis),” Laboratory Investigation 81, no. 12 (2001): 1581–1600. 11742030 10.1038/labinvest.3780373 PMC9827346

[50] A. Chahroudi , R. Chavan , N. Koyzr , E. K. Waller , G. Silvestri , and M. B. Feinberg , “Vaccinia Virus Tropism for Primary Hematolymphoid Cells Is Determined by Restricted Expression of a Unique Virus Receptor,” Journal of Virology 79, no. 16 (2005): 10397–10407. 16051832 10.1128/JVI.79.16.10397-10407.2005 PMC1182677

[51] D. A. Schwartz , et al., “Monkeypox Virus in Pregnancy, the Placenta and Newborn: An Emerging Poxvirus With Similarities to Smallpox and Other Orthopoxvirus Agents Causing Maternal and Fetal Disease,” Archives of Pathology & Laboratory Medicine 147, no. 7 (2023): 746–757, 10.5858/arpa.2022-0520-SA. 36857117

[52] S. Khattak , et al., “Monkeypox Virus Preparation in Pakistan‐Next Viral Zoonotic Disease Outbreak After COVID‐19,” Biomedical Letters 8, no. 2 (2022): 196–201.

[53] R. Vivancos , C. Anderson , P. Blomquist , et al., “Community Transmission of Monkeypox in the United Kingdom, April to May 2022,” Eurosurveillance 27, no. 22 (2022): 2200422. 35656834 10.2807/1560-7917.ES.2022.27.22.2200422 PMC9164677

[54] F. S. Minhaj , et al., Monkeypox Outbreak—Nine states, May 2022: Weekly/June 10, 2022/71 (23); 764–769. 2022, Wiley Online Library. 10.15585/mmwr.mm7123e1 PMC9181052 35679181

[55] N. Kumar , A. Acharya , H. E. Gendelman , and S. N. Byrareddy , “The 2022 Outbreak and the Pathobiology of the Monkeypox Virus,” Journal of Autoimmunity 131 (2022): 102855. 35760647 10.1016/j.jaut.2022.102855 PMC9534147

[56] M. J. Moore , B. Rathish , and F. Zahra , *Mpox (Monkeypox)*. 2021. 34662033

[57] V. S. Pandya , V. Mehta , M. Miraj , et al., “Monkeypox: An Unfamiliar Virus—Clinical and Epidemiological Characteristics, Diagnosis, and Treatment With Special Emphasis on Oral Health,” Diagnostics 12, no. 11 (2022): 2749. 36359593 10.3390/diagnostics12112749 PMC9689609

[58] M. B. Valli , A. Vulcano , M. Rueca , et al., “Concomitant Syndromic Diagnosis of Mpox and Other Vesicular Viruses in Patients With Skin and Genital Lesions,” Pathogens 13, no. 3 (2024): 207. 38535550 10.3390/pathogens13030207 PMC10974789

[59] B. M. Liu , N. Y. Rakhmanina , Z. Yang , and M. I. Bukrinsky , “Mpox (Monkeypox) Virus and Its Co‐Infection With HIV, Sexually Transmitted Infections, or Bacterial Superinfections: Double Whammy or a New Prime Culprit?,” Viruses 16, no. 5 (2024): 784. 38793665 10.3390/v16050784 PMC11125633

[60] M. A. Zinnah , M. B. Uddin , T. Hasan , et al., “The Re‐Emergence of Mpox: Old Illness, Modern Challenges,” Biomedicines 12, no. 7 (2024): 1457. 39062032 10.3390/biomedicines12071457 PMC11274818

[61] F. Branda , C. Romano , M. Ciccozzi , et al., “Mpox: An Overview of Pathogenesis, Diagnosis, and Public Health Implications,” Journal of Clinical Medicine 13, no. 8 (2024): 2234. 38673507 10.3390/jcm13082234 PMC11050819

[62] P. K. Mbala , J. W. Huggins , T. Riu‐Rovira , et al., “Maternal and Fetal Outcomes Among Pregnant Women With Human Monkeypox Infection in the Democratic Republic of Congo,” Journal of Infectious Diseases 216, no. 7 (2017): 824–828. 29029147 10.1093/infdis/jix260

[63] A. Hussain , J. Kaler , G. Lau , and T. Maxwell , “Clinical Conundrums: Differentiating Monkeypox From Similarly Presenting Infections,” Cureus 14, no. 10 (2022): e29929, 10.7759/cureus.29929. 36348880 PMC9634140

[64] E. Petersen , A. Kantele , M. Koopmans , et al., “Human Monkeypox: Epidemiologic and Clinical Characteristics, Diagnosis, and Prevention,” Infectious Disease Clinics of North America 33, no. 4 (2019): 1027–1043. 30981594 10.1016/j.idc.2019.03.001 PMC9533922

[65] A. MacNeil , M. G. Reynolds , Z. Braden , et al., “Transmission of Atypical Varicella‐Zoster Virus Infections Involving Palm and Sole Manifestations in an Area With Monkeypox Endemicity,” Clinical Infectious Diseases 48, no. 1 (2009): e6–e8. 19025497 10.1086/595552

[66] M. Altindis , E. Puca , and L. Shapo , “Diagnosis of Monkeypox Virus–An Overview,” Travel Medicine and Infectious Disease 50 (2022): 102459. 36109000 10.1016/j.tmaid.2022.102459 PMC9534096

[67] K. H. Hong , G. J. Kim , K. H. Roh , et al., “Guidelines for the Laboratory Diagnosis of Monkeypox in Korea,” Annals of Laboratory Medicine 43, no. 2 (2023): 137–144. 36281507 10.3343/alm.2023.43.2.137 PMC9618902

[68] A. M. McCollum and I. K. Damon , “Human Monkeypox,” Clinical Infectious Diseases 58, no. 2 (2014): 260–267. 24158414 10.1093/cid/cit703 PMC5895105

[69] B. Bížová , D. Veselý , M. Trojánek , and F. Rob , “Coinfection of Syphilis and Monkeypox in HIV Positive Man in Prague, Czech Republic,” Travel Medicine and Infectious Disease 49 (2022): 102368. 35661824 10.1016/j.tmaid.2022.102368 PMC9533838

[70] W. Bain , J. S. Lee , A. M. Watson , and M. S. Stitt‐Fischer , “Practical Guidelines for Collection, Manipulation and Inactivation of SARS‐CoV‐2 and COVID‐19 Clinical Specimens,” Current Protocols in Cytometry 93, no. 1 (2020): e77. 32502333 10.1002/cpcy.77 PMC7300551

[71] J. J. Sejvar , Y. Chowdary , M. Schomogyi , et al., “Human Monkeypox Infection: A Family Cluster in the Midwestern United States,” Journal of Infectious Diseases 190, no. 10 (2004): 1833–1840. 15499541 10.1086/425039

[72] E. Hammarlund , M. W. Lewis , S. V. Carter , et al., “Multiple Diagnostic Techniques Identify Previously Vaccinated Individuals With Protective Immunity Against Monkeypox,” Nature Medicine 11, no. 9 (2005): 1005–1011. 10.1038/nm1273 16086024

[73] Y. Li , V. A. Olson , T. Laue , M. T. Laker , and I. K. Damon , “Detection of Monkeypox Virus With Real‐Time PCR Assays,” Journal of Clinical Virology 36, no. 3 (2006): 194–203. 16731033 10.1016/j.jcv.2006.03.012 PMC9628957

[74] R. A. Maksyutov , E. V. Gavrilova , and S. N. Shchelkunov , “Species‐Specific Differentiation of Variola, Monkeypox, and Varicella‐Zoster Viruses by Multiplex Real‐Time PCR Assay,” Journal of Virological Methods 236 (2016): 215–220. 27477914 10.1016/j.jviromet.2016.07.024 PMC9629046

[75] U. Samarasekera , “WHO Ramps Up Emergency Use Mpox Diagnostics,” Lancet Microbe 6, no. 2 (2025): 101051. 39622260 10.1016/j.lanmic.2024.101051

[76] B. M. Liu and Z. Yang , “An Urgent Need for Diagnostic Tools to Address Global Mpox Public Health Emergencies,” Journal of Clinical Microbiology 63, no. 7 (2025): e01321‐24. 40470950 10.1128/jcm.01321-24 PMC12239723

[77] P.‐Y. Nguyen , W. S. Ajisegiri , V. Costantino , A. A. Chughtai , and C. R. MacIntyre , “Reemergence of Human Monkeypox and Declining Population Immunity in the Context of Urbanization, Nigeria, 2017–2020,” Emerging Infectious Diseases 27, no. 4 (2021): 1007. 33756100 10.3201/eid2704.203569 PMC8007331

[78] M. B. Townsend , M. S. Keckler , N. Patel , et al., “Humoral Immunity to Smallpox Vaccines and Monkeypox Virus Challenge: Proteomic Assessment and Clinical Correlations,” Journal of Virology 87, no. 2 (2013): 900–911. 23135728 10.1128/JVI.02089-12 PMC3554095

[79] M. Molero‐Abraham , J. P. Glutting , D. R. Flower , E. M. Lafuente , and P. A. Reche , “EPIPOX: Immunoinformatic Characterization of the Shared T‐Cell Epitome Between Variola Virus and Related Pathogenic Orthopoxviruses,” Journal of Immunology Research 2015 (2015): 1–11. 10.1155/2015/738020 PMC4641182 26605344

[80] R. B. Kennedy , I. G. Ovsyannikova , I. H. Haralambieva , D. E. Grill , and G. A. Poland , “Proteomic Assessment of Humoral Immune Responses in Smallpox Vaccine Recipients,” Vaccine 40, no. 5 (2022): 789–797. 34952760 10.1016/j.vaccine.2021.12.033 PMC8792332

[81] T. M. Mack , J. Noble, Jr. , and D. B. Thomas , “A Prospective Study of Serum Antibody and Protection Against Smallpox,” American Journal of Tropical Medicine and Hygiene 21, no. 2 (1972): 214–218. 5061278 10.4269/ajtmh.1972.21.214

[82] J. Nyame , S. Punniyakotti , K. Khera , R. S. Pal , N. Varadarajan , and P. Sharma , “Challenges in the Treatment and Prevention of Monkeypox Infection; a Comprehensive Review,” Acta Tropica 245 (2023): 106960. 37276922 10.1016/j.actatropica.2023.106960 PMC10239200

[83] A. Abdelaal , A. Reda , B. I. Lashin , et al., “Preventing the Next Pandemic: Is Live Vaccine Efficacious Against Monkeypox, or Is There a Need for Killed Virus and mRNA Vaccines?,” Vaccines 10, no. 9 (2022): 1419. 36146497 10.3390/vaccines10091419 PMC9500691

[84] P. E. M. Fine , Z. Jezek , B. Grab , and H. Dixon , “The Transmission Potential of Monkeypox Virus in Human Populations,” International Journal of Epidemiology 17, no. 3 (1988): 643–650. 2850277 10.1093/ije/17.3.643

[85] K. Kupferschmidt , “Monkeypox Vaccination Plans Take Shape Amid Questions,” Science 376, no. 6598 (2022): 1142–1143. 35679422 10.1126/science.add3743

[86] J. Reina and C. Iglesias , “Vaccines Against Monkeypox,” Medicina Clínica (English Edition) 160 (2023): 305–309. 37033199 10.1016/j.medcle.2023.01.005 PMC10037303

[87] G. Tiecco , M. Degli Antoni , S. Storti , L. R. Tomasoni , F. Castelli , and E. Quiros‐Roldan , “Monkeypox, a Literature Review: What Is New and Where Does This Concerning Virus Come From?,” Viruses 14, no. 9 (2022): 1894. 36146705 10.3390/v14091894 PMC9501516

[88] M. R. Islam , M. J. Hossain , A. Roy , et al., “Repositioning Potentials of Smallpox Vaccines and Antiviral Agents in Monkeypox Outbreak: A Rapid Review on Comparative Benefits and Risks,” Health Science Reports 5, no. 5 (2022): e798. 36032515 10.1002/hsr2.798 PMC9399446

[89] A. Y. Cheema , O. J. Ogedegbe , M. Munir , G. Alugba , and T. K. Ojo , “Monkeypox: A Review of Clinical Features, Diagnosis, and Treatment,” Cureus 14, no. 7 (2022): e26756, 10.7759/cureus.26756. 35967174 PMC9365327

[90] M. Reynolds , A. McCollum , B. Nguete , R. Shongo Lushima , and B. Petersen , “Improving the Care and Treatment of Monkeypox Patients in Low‐Resource Settings: Applying Evidence From Contemporary Biomedical and Smallpox Biodefense Research,” Viruses 9, no. 12 (2017): 380. 29231870 10.3390/v9120380 PMC5744154

[91] P. Conrad , “The Meaning of Medications: Another Look at Compliance,” Social Science & Medicine (1982) 20, no. 1 (1985): 29–37. 3975668 10.1016/0277-9536(85)90308-9

[92] J. P. S. Begum , L. Ngangom , P. Semwal , S. Painuli , R. Sharma , and A. Gupta , “Emergence of Monkeypox: A Worldwide Public Health Crisis,” Human Cell 36, no. 3 (2023): 877–893. 36749539 10.1007/s13577-023-00870-1 PMC9903284

[93] D. W. Grosenbach , R. Jordan , and D. E. Hruby , “Development of the Small‐Molecule Antiviral ST‐246® as a Smallpox Therapeutic,” Future Virology 6, no. 5 (2011): 653–671. 21837250 10.2217/fvl.11.27 PMC3151656

[94] R. Lanier , L. Trost , T. Tippin , et al., “Development of CMX001 for the Treatment of Poxvirus Infections,” Viruses 2, no. 12 (2010): 2740–2762. 21499452 10.3390/v2122740 PMC3077800

[95] A. T. Russo , D. W. Grosenbach , T. L. Brasel , et al., “Effects of Treatment Delay on Efficacy of Tecovirimat Following Lethal Aerosol Monkeypox Virus Challenge in Cynomolgus Macaques,” Journal of Infectious Diseases 218, no. 9 (2018): 1490–1499. 29982575 10.1093/infdis/jiy326 PMC6151088

[96] E. Sbrana , R. Jordan , D. E. Hruby , et al., “Efficacy of the Antipoxvirus Compound ST‐246 for Treatment of Severe Orthopoxvirus Infection,” American Journal of Tropical Medicine and Hygiene 76, no. 4 (2007): 768–773. 17426185

[97] CDC, C ., *Treatment Information for Healthcare Professionals*. 2023.

[98] G. Yang , D. C. Pevear , M. H. Davies , et al., “An Orally Bioavailable Antipoxvirus Compound (ST‐246) Inhibits Extracellular Virus Formation and Protects Mice From Lethal Orthopoxvirus Challenge,” Journal of Virology 79, no. 20 (2005): 13139–13149. 16189015 10.1128/JVI.79.20.13139-13149.2005 PMC1235851

[99] National Institute of Diabetes and Digestive and Kidney Diseases , *LiverTox: Clinical and Research Information on Drug‐Induced Liver Injury*. 2012: National Institute of Diabetes and Digestive and Kidney Diseases. 31643176

[100] E. R. Kern , C. Hartline , E. Harden , et al., “Enhanced Inhibition of Orthopoxvirus Replication In Vitro by Alkoxyalkyl Esters of Cidofovir and Cyclic Cidofovir,” Antimicrobial Agents and Chemotherapy 46, no. 4 (2002): 991–995. 11897580 10.1128/AAC.46.4.991-995.2002 PMC127114

[101] Y. Xu , Y. Wu , X. Wu , et al., “Structural Basis of Human Mpox Viral DNA Replication Inhibition by Brincidofovir and Cidofovir,” International Journal of Biological Macromolecules 270 (2024): 132231. 38735603 10.1016/j.ijbiomac.2024.132231

[102] I. Harrison , K. DeSear , B. A. Santevecchi , et al., “Brincidofovir for Disease Progression Due to Suspected Tecovirimat Resistance in Association With Advanced HIV,” International Journal of STD & AIDS 35, no. 8 (2024): 651–653. 38502040 10.1177/09564624241238813

[103] C. A. Contag , L. Mische , I. Fong , et al., “Treatment of Mpox With Suspected Tecovirimat Resistance in Immunocompromised Patient, United States, 2022,” Emerging Infectious Diseases 29, no. 12 (2023): 2520. 37856215 10.3201/eid2912.230849 PMC10683816

